# *In vitro* fermentation characteristics and prebiotic activity of herbal polysaccharides: a review

**DOI:** 10.3389/fnut.2025.1687766

**Published:** 2025-11-17

**Authors:** Yong Lai, Yu Wang, Chang Liu, Guanhua Lou, Jianan Feng, Ying Li

**Affiliations:** Institute of Traditional Chinese Medicine of Sichuan Academy of Chinese Medicine Sciences, Chengdu, Sichuan, China

**Keywords:** dietary fiber, gut microbiota, short chain fatty acid, *in vitro* fermentation, prebiotic, functional food

## Abstract

Herbal polysaccharides (HPs), as a category of macromolecular compounds, exhibits unique prebiotic benefits, including antioxidant and immunomodulatory effects, and regulatory effects on the gut microbiota. Particularly, the gut microbiota, often referred to as the ‘forgotten organ’ and the ‘second human genome’, plays a pivotal role in human health. *In vitro* fermentation techniques, including simulated gastrointestinal digestion and fermentation models, have emerged as effective tools for studying the gut microbiota and its relation to diseases. *In vitro* fermented polysaccharides can mimic the intestinal environment *in vivo*, which is crucial for understanding the mechanisms of polysaccharide metabolism, clarifying their metabolic pathways, and elucidating their prebiotic activity. By integrating high-impact research from the past 5 years, this review examines the structural changes of HPs during *in vitro* fermentation, their microbial metabolic mechanisms, and their prebiotic activity, addressing a current gap by integrating the ‘structure-microbe-function’ relationship within this field. The findings provide a theoretical basis for the effective utilization of traditional herbs, and offer insights for the development of novel functional products. Future studies should combine dynamic fermentation models with stratified clinical trials to accelerate the translational application of herbal polysaccharides.

## 1 Introduction

Herbal plants, revered since antiquity as a cornerstone of global ethnomedicine, constitute an invaluable biocultural heritage (1, 2). Herbs of different origins, structures and forms are rich in a variety of active ingredients, including polysaccharides, polyphenols, flavonoids, saponins, alkaloids and volatile oils (3–5). Notably, polysaccharide-rich herbs usually have excellent antioxidant, hypoglycemic, hypolipidemic, immunomodulatory, and gut microbiota-regulating properties (6, 7). As an important class of biomolecules, polysaccharides are widely found in various types of organisms and play a key role in life activities (8). With the continuous development of sugar science in recent years, polysaccharides have shown great value in food, medicine, cosmetics and other industries. Especially in the pharmaceutical field, herbal polysaccharides (HPs) have become an important raw material for the research and development of new drugs and health products due to their rich biological activity (9, 10).

As a key component of the human microecosystem, gut microbiota is of great significance to host health (11). It is involved in many physiological processes such as food digestion, nutrient absorption, immune regulation, and its imbalance is closely related to the development of a variety of diseases such as obesity, diabetes mellitus, cardiovascular disease and neurodegenerative diseases (12, 13). Studies have shown that polysaccharides and gut microbiota have a complex and mutual relationship. On the one hand, the gut microbiota can metabolize polysaccharides, produce short-chain fatty acids (SCFAs) and other beneficial metabolites, which can regulate intestinal pH, promote intestinal peristalsis, and also enhance immunity by regulating immune cell function (14). On the other hand, polysaccharides can be used as prebiotics to selectively promote the growth and reproduction of beneficial bacteria while inhibiting the growth of harmful bacteria, thus maintaining the balance of gut microbiota (15).

*In vitro* fermentation of polysaccharides is a process of *in vitro* using microorganisms that mimic the intestinal environment *in vivo*. This process is usually carried out in a fermentation system containing a specific microbial community, and the metabolic changes of polysaccharides under the action of microorganisms are investigated by controlling the conditions such as temperature, pH, and substrate concentration (16, 17). *In vitro* fermentation is of great significance, as it helps to understand the metabolic mechanism of polysaccharides in the intestinal tract, clarifies the pathways and products of polysaccharides utilized by microorganisms, and provides a theoretical basis for the interpretation of polysaccharides’ prebiotic activity and their effects on intestinal health (18, 19). In addition, *in vitro* fermentation technology has become an effective means of screening polysaccharides with specific functions due to its highly controllable and reproducible nature (20).

Therefore, this review aims to explore the *in vitro* fermentation characteristics and prebiotic potential of HPs. It focuses on summarizing the metabolic processes of HPs in simulated *in vitro* fermentation, their effects on microbial communities, and the associated biological effects. By investigating various *in vitro* fermentation models of different polysaccharides and identifying those with potential prebiotic and immunomodulatory activities, this review provides a theoretical foundation for the development of novel functional products.

## 2 Review methodology

This narrative review was designed to rapidly map the recent landscape of HP in-vitro fermentation research rather than to perform a quantitative meta-analysis. To ensure transparency we nevertheless adopted a structured search strategy. Web of Science Core Collection and PubMed were interrogated from January 2014 to October 2024 (last search 20 October 2024) with the following string: (herbal polysaccharide* OR “plant polysaccharide*” OR “medicinal mushroom polysaccharide*”) AND (“*in vitro* fermentation” OR “fecal fermentation” OR “gut microbiota fermentation”) AND (SCFA* OR “short-chain fatty acid*” OR “prebiotic*” OR “microbiome”), restricted to English-language articles published in JCR Q1–Q3 journals. After duplicate removal, full-text evaluation yielded 134 eligible studies, including 41 highly cited or hotspot papers. Due to the evident methodological heterogeneity, the data are descriptive rather than statistical. Additionally, this review confirms that all evidence is based on *in vivo* animal studies with appropriate controls or *in vitro*/ex vivo experiments of equivalent quality.

## 3 Acquisition of HPs in fermentation and their relation to gut microbiota

### 3.1 Chemical structure and classification of HPs

In the early days, people discovered the medicinal value of certain HPs through traditional medicine and practical experience (21), and used them for disease treatment, which provided important directions for subsequent scientific exploration (22, 23). With the development of science and technology, researchers have employed chemical analytical methods to separate and identify polysaccharides, gradually clarifying their chemical structures and compositions (24). Studies on the polysaccharides of common herbal plants have revealed their structural characteristics, including monosaccharide composition and glycosidic bond types (25). The chemical structure of HPs is complex and diverse, primarily composed of monosaccharides linked by glycosidic bonds (26). Factors such as monosaccharide composition, glycosidic bond types, degree of branching, and molecular weight collectively determine the structure and function of polysaccharides. Based on monosaccharide composition, HPs can be classified into homopolysaccharides and heteropolysaccharides. Homopolysaccharides are composed of a single type of monosaccharide, such as starch, which is polymerized from glucose monomers; heteropolysaccharides consist of two or more types of monosaccharides (27–29). From a functional perspective, they can be divided into storage polysaccharides and structural polysaccharides, with the former exemplified by starch and the latter by cellulose in plant cell walls (30). HPs with different structures exhibit diverse biological activities. Polysaccharides with specific glycosidic bonds and branching structures may be more readily recognized and utilized by gut microbiota, thereby exerting prebiotic activity (31). In contrast, certain polysaccharides containing special functional groups may possess stronger antioxidant and immunomodulatory activities. Accumulating evidence indicates that the biological activity of polysaccharides is closely linked to their structural characteristics, particularly the types of functional groups (sulfate, acetyl, carboxyl, amino) which significantly influence antioxidant and immunomodulatory activities (32). For instance, sulfated polysaccharides (such as those derived from certain algae or fungi) exhibit stronger antioxidant activity due to their sulfate groups enhancing free radical scavenging capacity (33). Furthermore, acetylation modifications can enhance immunomodulatory activity by altering the polysaccharide’s hydrophobicity and spatial conformation, thereby improving its binding affinity to immune cell surface receptors (34).

### 3.2 Extraction, isolation and purification of HPs

The extraction and purification of HPs are crucial for obtaining high purity and high activity polysaccharides. Common extraction methods include water extraction, acid extraction, alkali extraction, enzymatic extraction, ultrasound - assisted extraction, and microwave - assisted extraction (35–37). Water extraction is simple and low-cost but may have low efficiency. Acid and alkali extraction can increase the extraction rate but may damage the polysaccharide structure. Enzymatic extraction is mild and specific, avoiding polysaccharide structural damage. Ultrasound and microwave - assisted methods accelerate polysaccharide release through physical effects, improving extraction efficiency (38). Purification often involves column chromatography, ultrafiltration, and precipitation. Column chromatography, such as ion - exchange and gel - permeation chromatography, separates and purifies polysaccharides based on charge and molecular weight (39). Ultrafiltration uses suitable membranes to remove impurities, and precipitation methods like ethanol precipitation achieve preliminary purification (40). Wu et al. demonstrated that compared to traditional hot water extraction of Acidic Polysaccharides from Lotus Leaves, deep eutectic solvent-assisted extraction (LLP-D) reduced the molecular weight of the main component from 12.9 × 10^4^ Da to 4.0 × 10^4^ Da and significantly increased the esterification degree. These structural alterations rendered LLP-D more readily fermentable by gut microbiota *in vitro*, as evidenced by substantial enhancements in antioxidant, hypoglycemic, and immunostimulatory activities (41). These studies demonstrate that extraction processes not only determine polysaccharide yield but also enhance fermentability and functional activity through a dual mechanism of “shearing-functional group modification”.

In identification, chromatography and spectrometry are widely used. Size - exclusion chromatography (SEC) and asymmetric flow field - flow fractionation (AF4) separate polysaccharides by hydrodynamic volume (42, 43). Combined with multi - angle laser light scattering (MALLS) and refractive index (RI) detectors, they provide information on molecular weight distribution, size, branching, and conformation (44). Matrix-assisted laser desorption/ionization-time-of-flight mass spectrometry (MALDI-TOF MS) analyzes oligosaccharides released after periodate oxidation and hydrolysis, distinguishing different polysaccharides (45, 46). These technologies have greatly advanced the study of HPs’ structure and function. Combining different extraction and purification techniques can effectively enhance the purity and activity of HPs.

### 3.3 Mechanism of HPs fermentation by gut microbiota

**Figure 1:**
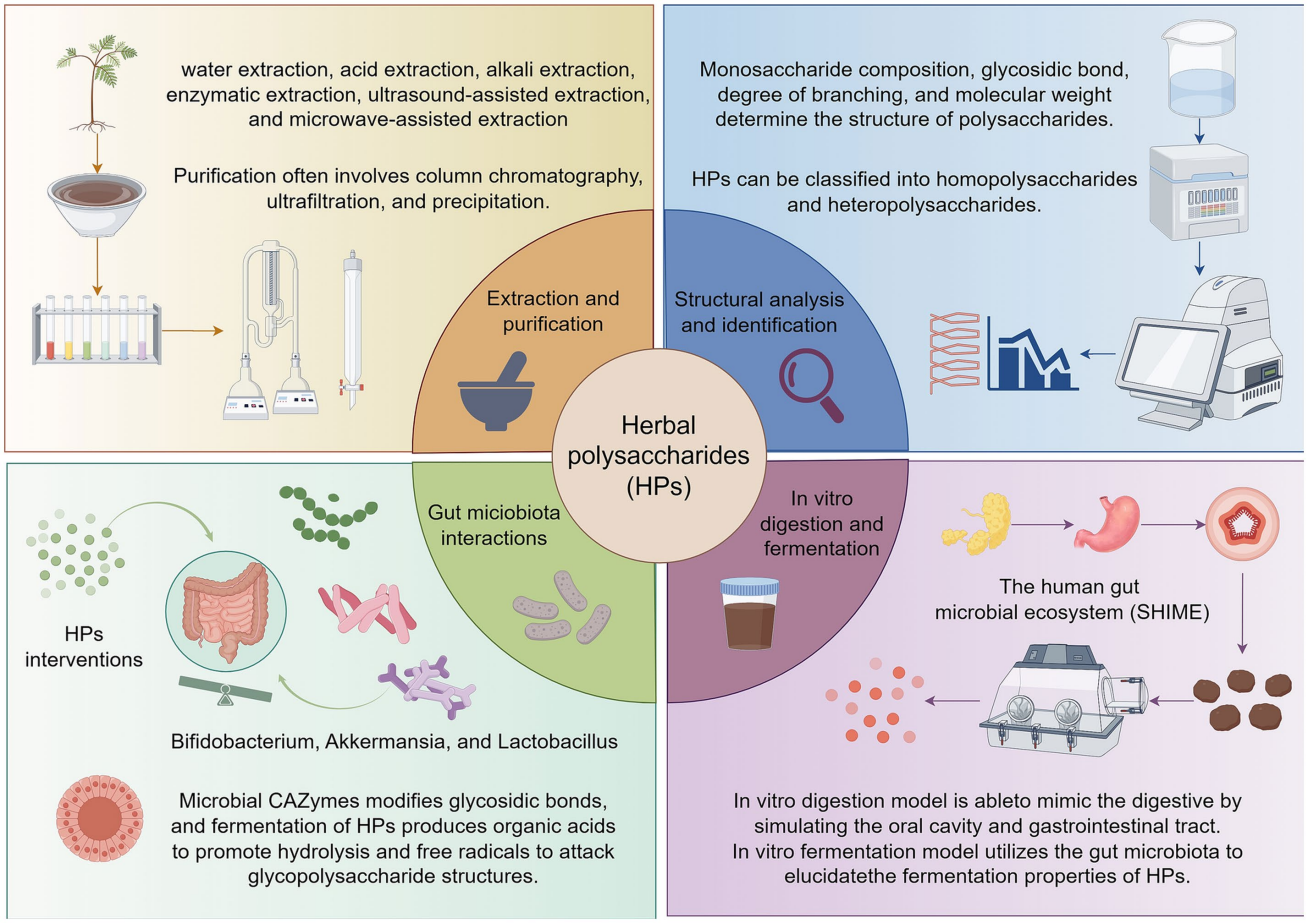
Overview of the extraction, structural characterization, and in vitro digestion-fermentation processes of HPs. Herbal polysaccharides are extracted using various methods including water, acid, alkali, enzymatic, ultrasound-assisted, and microwave-assisted extraction. These polysaccharides are primarily composed of monosaccharides linked by glycosidic bonds, with structural diversity influenced by monosaccharide composition, glycosidic bond types, degree of branching, and molecular weight. In vitro digestion and fermentation models are essential tools for evaluating the functional properties of HPs. The simulated gastrointestinal digestion model mimics the oral, gastric, and intestinal phases of human digestion, allowing for the assessment of structural changes and digestibility. The in vitro fermentation model utilizes gut microbiota to investigate the metabolic fate of HPs, including their effects on microbial composition and metabolite production. Created by Figdraw.com.

Numerous studies have shown that HPs possess a wide range of biological activities, including anti-inflammatory, anti-tumor, immunomodulatory, hypoglycemic and hypolipidemic biological activities, and exhibit broad application prospects in the fields of medicine and food (Figure 1) (47). Microbial fermentation can significantly influence polysaccharide purity while regulating their structure, thereby exerting a substantial impact on their biological activity (48). On one hand, fermentation degrades non-sugar impurities such as proteins, lipids, and pigments in plant raw materials, indirectly enhancing the relative purity of polysaccharides (49). On the other hand, microorganisms may synthesize new metabolites during their metabolic processes, such as extracellular polysaccharides, pigments, or organic acids (50, 51). The interaction of HPs with specific microorganisms or gut microbiota during fermentation is the central mechanism for the structural and functional changes of the polysaccharides (52). The secretion of CAZymes, the production of organic acids and the attack of free radicals work together to determine the final properties of HPs during fermentation (53). First, microorganisms secrete carbohydrate-active enzymes (CAZymes) that alter the structure and function of HPs by specifically hydrolyzing or modifying glycosidic bonds (54). The CAZymes family consists of glycoside hydrolases, polysaccharide cleaving enzymes, and pectin methylesterases, among others, whose activity is influenced by fermentation pH, temperature, and substrate properties. These enzymes reduce the molecular weight of polysaccharides by cleaving glycosidic bonds or removing side-chain groups, altering their solubility, viscosity and biological activity (55, 56). Secondly, organic acids (lactic acid, acetic acid) produced by bacterial fermentation promote the hydrolysis of HPs by lowering the environmental pH, altering their molecular weight and functional properties (57). Different microorganisms produce different types of organic acids, resulting in polysaccharides exhibiting different structural and functional changes during fermentation (58). Additionally, free radicals generated during fermentation lead to polysaccharide breakage and structural changes by attacking either glycosidic bonds or side chains groups of polysaccharides (59). Free radical action may expose additional active sites that enhance the antioxidant or immunomodulatory capacity of polysaccharides, and may also lead to cross-linking of polysaccharides to form more stable network structures (60, 61). At the molecular level, metagenomic evidence from *Bacteroides ovatus* suggests that the loci Bov_0483–0486 encode enzymes able to cleave (1–5)-*α*-L-Araf linkages, while NADH-oxidase-generated radicals have been proposed (but not yet directly demonstrated in herbal-polysaccharide fermentation) to introduce aldehyde groups that may contribute to antioxidant activity (62, 63).

### 3.4 *In vitro* digestion characteristics of HPs

In vitro modeling is an important tool for studying the biological activity and mechanism of action of HPs. Commonly used *in vitro* models include simulated gastrointestinal digestion model and in vitro fermentation model (64). Among them, the simulated gastrointestinal tract digestion model is able to dynamically track the structural and property changes of HPs at different stages of digestion by simulating the digestive environments of the oral cavity, stomach and small intestine (65). It was found that the digestive behaviors of different HPs in the gastrointestinal tract were significantly different: some polysaccharides were depolymerized in the gastric acid environment, the molecular weight was reduced, and the content of reducing sugars was significantly increased; while others could pass through the gastrointestinal tract in one piece and finally reach the large intestine (66, 67).

**Figure 2:**
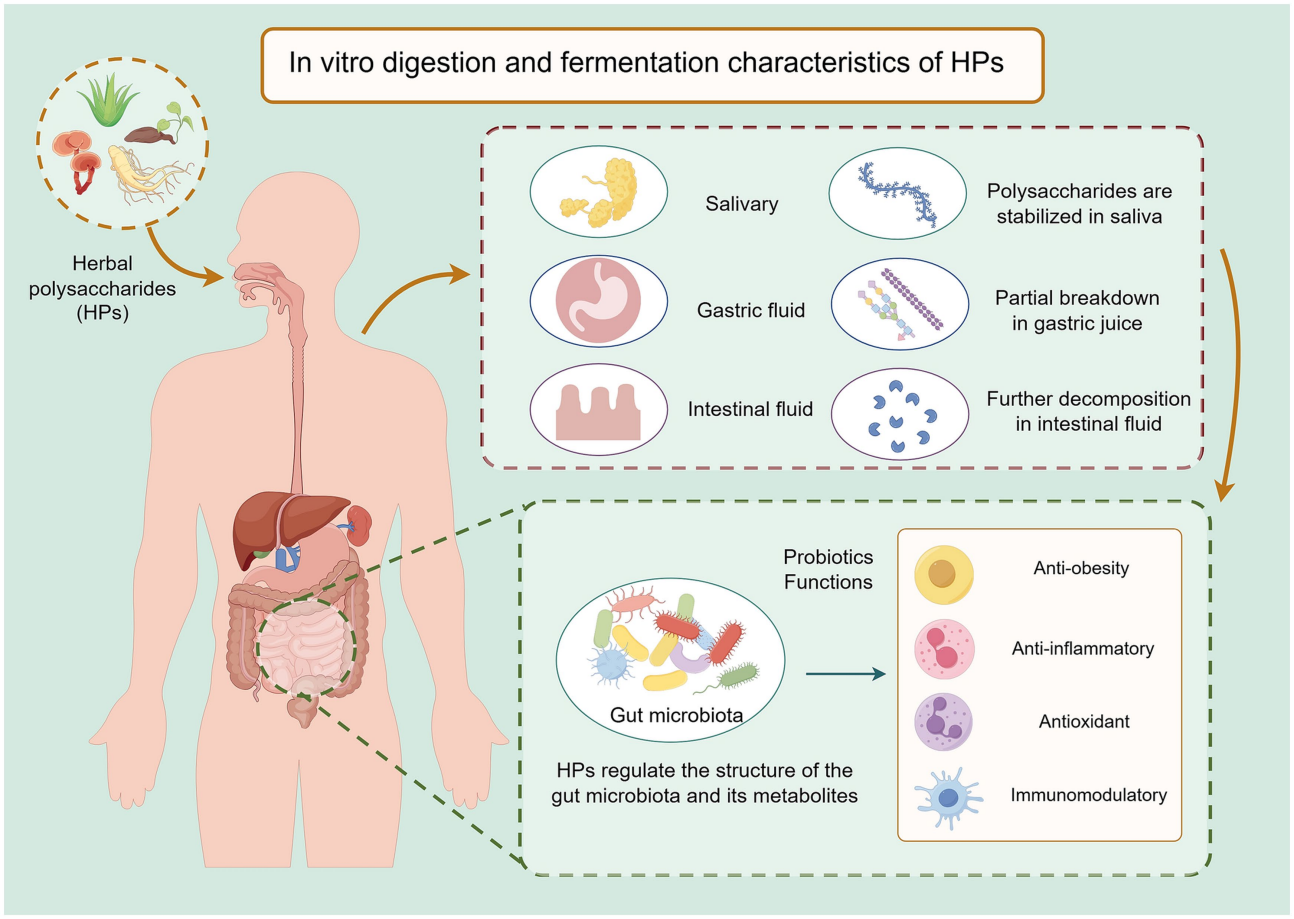
Schematic illustration of the enhancement of biological activity of HPs during in vitro digestion and fermentation. During digestion, HPs undergo partial depolymerization in the gastric and intestinal environments, leading to reduced molecular weight and increased solubility. Subsequent fermentation by gut microbiota further degrades HPs into smaller oligosaccharides or monosaccharides, and promotes the production of beneficial metabolites such as SCFAs. These structural and metabolic transformations not only improve the bioaccessibility of HPs but also expose additional functional groups, thereby amplifying their antioxidant, anti-inflammatory, and immunomodulatory properties. Created by Figdraw.com.

Specifically, salivary amylase hydrolyzes most HPs weakly in the oral phase, and thus these polysaccharides usually enter the stomach intact (68). In the stomach, the synergistic effect of the gastric acid environment and pepsin may lead to depolymerization of some polysaccharides, but this process varies depending on the polysaccharide species. The type of glycosidic bond, degree of branching, molecular weight distribution, and presence of substituent groups of polysaccharides are closely related to the digestive process (69, 70). For example, *α*-glycosidic bonds are more easily hydrolyzed by human digestive enzymes than *β*-glycosidic bonds, while highly branched structures or tight folding of polysaccharide chains may enhance their resistance to digestion (71). Studying the *in vitro* digestion process of HPs not only helps to reveal their metabolic fate in the human body, but also provides a scientific basis for evaluating their nutritional value, biological activity and potential functional applications (Figure 2).

*In vitro* digestion and fermentation models provide rapid, controlled, and ethically acceptable preliminary screening for how HPs are metabolized by the human gut microbiota. However, they cannot replicate the dynamic absorption, mucus layer, host immune feedback, or circadian variability present in the human gut. Therefore, the antitumor, anti-obesity, and immunomodulatory effects demonstrated in *in vitro* studies are considered preliminary. All in vitro fermentation data must undergo sequential validation in animal models, pharmacokinetic studies, and stratified human trials before translating to clinical outcomes.

## 4 *In vitro* fermentation characteristics of HPs

**Figure 3:**
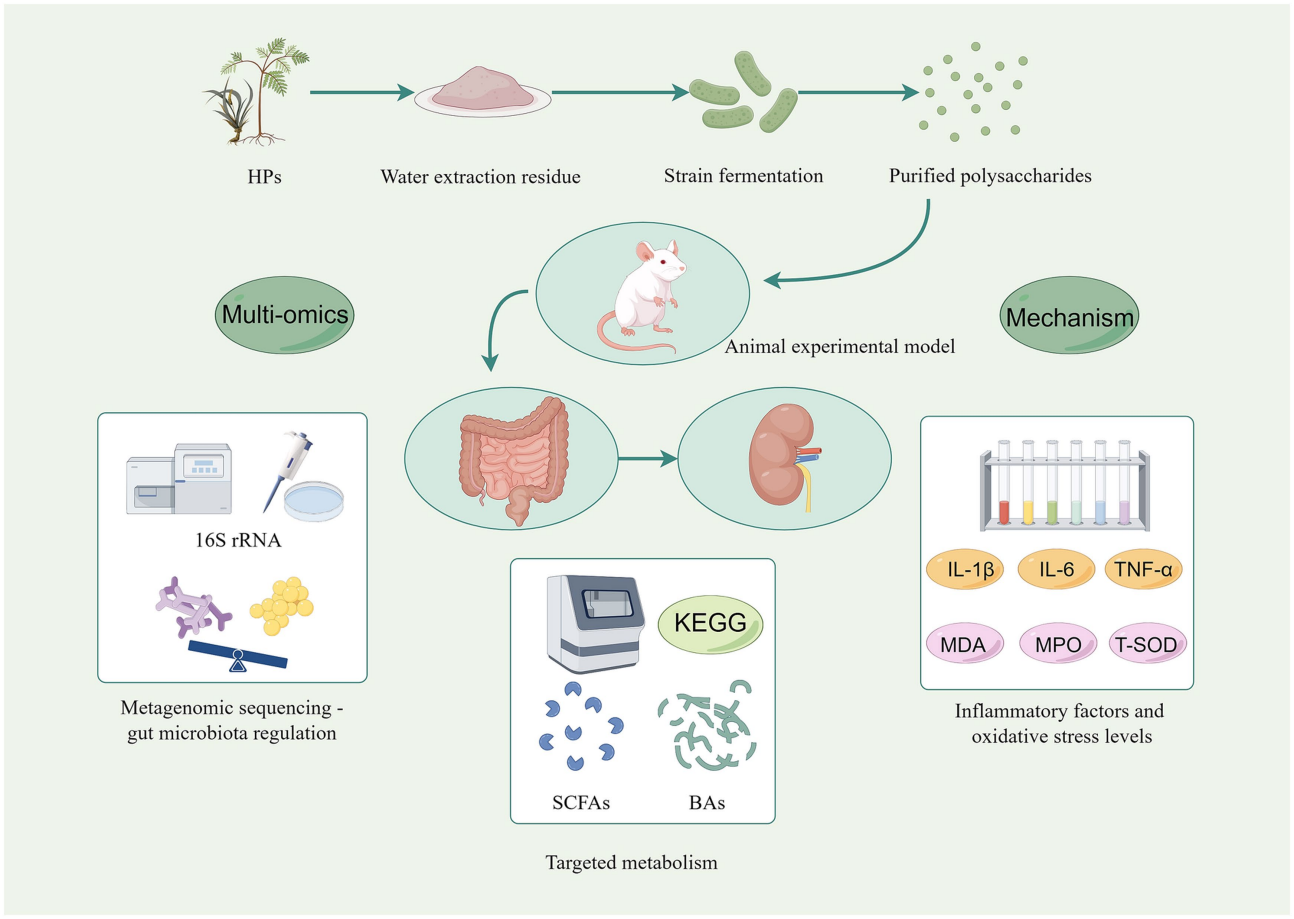
Multi-omics framework for investigating the functional transformation of HPs through microbial fermentation. Water-extracted HPs is subjected to either monostrains (*Lactobacillus, Bacteroides*) or complex fecal inocula under anaerobic in vitro conditions. Multi-omics tools are then integrated: 16S rRNA and metagenomic sequencing track real-time shifts in microbial community structure and CAZymes; targeted metabolomics quantify SCFAs, bile acids and oxidative-stress markers (MDA, MPO, T-SOD); transcriptomics/proteomics link HP-derived metabolites to host epithelial responses (IL-1*β*, IL-6, TNF-*α*, ZO-1, occludin). Created by Figdraw.com.

In vitro fermentation modeling utilizes intestinal microbiota to elucidate the fermentation properties of polysaccharides and their subsequent effects on the gut microbiota (Figure 3). This experimental approach enables the investigation of polysaccharide-induced changes in SCFA production and microbial community structure, thereby providing a foundation for understanding the metabolism of HPs within the gastrointestinal tract and their prebiotic activity. When assessing the prebiotic potential of HPs, the design of *in vitro* fermentation experiments is of paramount importance (72). Specifically, various types and concentrations of HPs are typically employed as fermentation substrates, with blank control groups and positive control groups supplemented with known prebiotics (inulin or oligofructose) being established during the experimental process. The fermentation characteristics and their impact on the metabolic activity of the intestinal microbiota can be systematically evaluated by monitoring key fermentation indicators, such as pH, SCFA content, and gas production (73). Concurrently, high-throughput 16S rRNA gene sequencing or metagenomic sequencing can be utilized to comprehensively analyze the effects of polysaccharides on the growth and abundance of diverse bacterial populations (74).

### 4.1 Fermentation characteristics of HPs with special strains

During *in vitro* fermentation of HPs, specific strains such as *Bifidobacterium, Lactobacillus*, *Akkermansia muciniphila*, and *Roseburia* exhibit significant polysaccharide degradation capacity (75, 76). These strains promote the production of SCFAs by secreting enzyme systems such as polysaccharide lyase and glycoside hydrolase, which degrade complex polysaccharides into low molecular weight fragments or monosaccharides (77). This fermentation pathway not only enhances the prebiotic function of polysaccharides, but also significantly improves the immunomodulatory potential of polysaccharides by regulating the structure of the gut microbiota and the production of metabolites (Table 1).

**Table 1:** HPs fermented by specific strains and their biological activity.

Herbs	Polysaccharides	Molecular weight		Monosaccharide	Fermented bacteria	Bioactivity	Reference
		Pre-fermentation	Post-fermentation				
*Tetrastigma hemsleyanum Diels et Gilg*	F-THDP2	3,580 kDa	1,230 kDa	glucose, galactose, and mannose at a ratio of 9.5:4.1:1.1	*Sanghuangporus sanghuang*	F-THDP2 induced apoptosis in HeLa cells through the Fas/FasL-mediated caspase-3 signaling pathway and exhibited superior antitumor activity.	(78)
*longan pulp*	LP-F	221.63 kDa	109.62 kDa	arabinose, xylose, mannose, glucose and galactose	*Lactobacillus*	LP-F was able to stimulate the secretion of NO and IL-6 from RAW264.7 macrophages more efficiently and showed stronger promotion of the proliferation of *Leuconostoc mesenteroides* and *Lactobacillus casei.*	(79)
*Polygonatum kingianum*	PKPS	50–650 kDa	2–100 kDa	Glucose, fructose, galacturonic acid, galactose, xylose, fucose, arabinose, glucuronic acid and mannose	*Lactobacillus paracasei*	Fermented PKPS showed higher DPPH radical scavenging and reducing power, 35.52% and 4.34-fold higher inhibitory activity of PS2-2 on α-glucosidase and lipase, respectively, reduced β-galactosidase activity, and inhibited secretion of SASP factors	(80)
*Flammulina velutipes*	FVP	9,520 kDa	9,460 kDa	glucosamine, galactose, glucose, mannose and glucuronic acid, with a molar ratio of about 4.88:10.24:72.94:5.47:6.47	*Bacteroides*	FVP fermentation increased beneficial bacteria (*Bacteroides, Parabacteroides, Catenibacterium,* and *Enterococcus*) while inhibiting the growth of harmful bacteria (*Escherichia-Shigella, Alistipes, Klebsiella,* and *Faecalibacterium*), and increased SCFAs.	(81)
*Brassica rapa L.*	BRP2–2	33.9 kDa	N	galacturonic acid, rhamnose, glucuronic acid, galactose and arabinose with a ratio of 87.17:4.44:2.02:3.69:2.67.	*Bacteroides ovatus*	BRP2-2 up-regulated 143 genes, including five independent polysaccharide utilization sites (PULs) and two carbohydrate-active enzymes (CAZymes) clusters, by regulating the structure of the gut microbiota and promoting the production of beneficial metabolites	(82)
*Litchi pulp*	LPF	2225.8 kDa	1743.9 kDa	arabinose, galacturonic acid, galactose, glucuronic acid, glucose and mannose	*Lactobacillus fermentum CICC 21828*	LPF promoted the proliferation of Megamonas, Prevotella and Bacteroides during the fermentation process and produced higher acetic acid and butyric acid, showing better fermentation characteristics and prebiotic activity.	(83)
*Nostoc commune Vauch*	NCVP	501.267 kDa	257.883 kDa	D-glucosamine, arabinose, galactose, glucose, xylose, mannose and glucuronic acid	*L. rhamnosus LR1, L. rhamnosus LR2 and L. plantarum LP1*	The antioxidant activity of NCVP was significantly increased after digestion in vitro, and the fermentation produced a variety of SCFAs that promoted the proliferation of *Bifidobacterium, Megamonas, Prevotella,* and *Bacteroides*.	(84)

An *in vitro* study found that the monosaccharide composition of *Tetrastigma hemsleyanum* polysaccharide (THDP2) shifted to galactose: mannose: fucose (12.6:2.3:1.0) after fermentation in *Sanghuangporus* sanghuang, with a significant decrease in molecular weight to 1,230 kDa, and the main chain structure was transformed to *α*-1,2-D-galactose and α-1,2-D-mannose. This structural reprogramming significantly modulated tumor metabolites, suggesting that the fermentation process may activate the immunomodulatory pathway by exposing more bioactive sites or changing the conformation (78). Another study showed that *longan* fruit pulp polysaccharide (LP) was fermented to increase solubility by 86.59% and decrease apparent viscosity by 81.67%, while enhancing immunomodulatory and prebiotic activities. This change was closely related to the degradation of the polysaccharide molecular chain, and the low molecular weight fragments produced during fermentation were more easily utilized by the gut microbiota, thus promoting the generation of SCFAs (79). Furthermore, *Polygonatum kingianum* polysaccharide (PKPS) fermented by *Lactobacillus paracasei* showed that the low molecular weight fractions PS2-4 (10–50 kDa) prolonged the lifespan by 20.70% and significantly enhanced DPPH free radical scavenging and *α*-glucosidase inhibitory activities in the *Cryptobacterium hidradenii* nematode model (80). This suggests that fermentation may activate the antioxidant, hypoglycemic and anti-aging functions of polysaccharides by introducing new functional groups or changing the conformation. *Flammulina velutipes* polysaccharides (FVP), as heteropolysaccharides with molecular weights up to 9.52 × 10^3^ kDa, exhibited significant anti-degradation properties in *in vitro* fermentation, while demonstrating intestinal health protective effects by regulating the composition of gut microbiota to promote *bifidobacteria*l and lactic acid bacteria proliferation and metabolite profiles (81).

Interestingly, several studies have collectively shown that the fermentation process not only changes the molecular structure of polysaccharides, but polysaccharide degradation is synergistically utilized with commensal bacteria and activity is enhanced in a coupled digestion-fermentation system. *Brassica rapa* L. polysaccharide (BRP2-2) showed a significant decrease in molecular weight within 24 h of *in vitro* fermentation, suggesting that it is efficiently utilized by intestinal microorganisms. BRP2-2 revealed the molecular interaction mechanism between microorganisms and polysaccharides by up-regulating 143 genes (five independent polysaccharide utilization sites, PULs, and two clusters of carbohydrate-activating enzymes, CAZymes) in response to BRP2-2-induced degradation processes (82). Similarly, *Lactobacillus fermentum* polysaccharides (LPF) pretreated with *Lactobacillus fermentum* decreased the molecular weight and promoted the utilization efficiency of *Bifidobacterium, Megalobacterium,* and *Prevotella* while producing higher concentrations of acetic acid and butyric acid. This synergistic effect suggests that fermentation lowers the threshold of polysaccharide utilization and optimizes the metabolic network of the bacteria (83). Furthermore, the presence of glucosamine (GlcN) in the monosaccharide composition of *Nostoc commune* Vauch. polysaccharides (NCVP) after *in vitro* digestion was hypothesized to be related to the synergistic effect of HCl and digestive enzymes. SCFAs produced by subsequent fermentation further promoted the proliferation of *Bifidobacterium* and *Bacteroidetes*, suggesting that the coupled process of digestion and fermentation may enhance the antioxidant and prebiotic activity of polysaccharides by exposing hidden active moieties or generating novel oligosaccharides (84).

In addition, with the widespread application of omics technologies such as metagenomics, metabolomics, and transcriptomics, the strain-specific functions of beneficial bacteria like *Lactobacillus* and *Bifidobacterium*. in fermented foods have gradually been revealed (85). Through metagenomic analysis of 19,699 food-derived LAB (FLAB) strains globally, Jin et al. identified significant differences in amino acid synthesis, SCFA metabolism, and secondary metabolite production among strains belonging to genera such as *Limosilactobacillus, Lactococcus,* and *Streptococcus* (86). Furthermore, Wu et al. employed a multi-omics approach to reveal that *Limosilactobacillus reuteri* synergistically interacts with galacto-oligosaccharides (GOS) to enrich *Bacteroides acidifaciens* and promote its synthesis of pentadecanoic acid (87). This process inhibits the NF-κB signaling pathway and enhances tight junction protein expression. Multi-omics evidence has clearly demonstrated that the enrichment of beneficial bacteria can translate into health benefits for the host.

### 4.2 Fermentation characteristics of HPs from animal manure

#### 4.2.1 Fermented HPs are accompanied by a decrease in molecular weight

During *in vitro* fermentation, the complex interactions between animal fecal microbial communities and plant-derived polysaccharides are a key link in the regulation of intestinal microecology and host health. Polysaccharides synergistically enhance their antioxidant, anti-inflammatory and prebiotic potentials through multiple pathways during fermentation, including dynamic adjustment of molecular weight, structural optimization of the gut microbiota, and precise regulation of SCFAs (88). HPs are often accompanied by significant changes in molecular weight during *in vitro* fermentation. Polysaccharides undergo depolymerization and rearrangement under the action of microbial enzymes, and the decrease in molecular weight not only increases their accessibility, but also exposes potential bioactive sites, laying a structural foundation for subsequent functional activation (Table 2). Interestingly, existing research indicates that the effects of microbial fermentation on HPs extend beyond mere molecular weight reduction (89). In certain cases, microorganisms may secrete extracellular polysaccharides (EPS) during fermentation or repolymerize low-molecular-weight sugar units through mechanisms such as glycosyltransferases, resulting in increased polysaccharide molecular weight (90).

**Table 2:** HPs from animal manure fermentation and their biological activity.

Plants	Polysaccharides	Molecular weight	Bioactive functions	Gut microbiota regulation	Gut metabolites regulation	Reference
*Oudemansiella radicata*	ORP	lower digestibility during in vitro simulated digestion, with a slight decrease in molecular weight from 39.5 kDa to 34.3 kDa	the antioxidant activity of ORP was significantly enhanced	ORP significantly increased the relative abundance of Bacteroides and Parabacteroides while decreased the ratio of Firmicutes/Bacteroidetes	Acetic acid, propionic acid, and butyric acid were the major short-chain fatty acids (SCFAs) products during in vitro fermentation	(91)
*kiwifruit pomace*	KFP	The molecular weight of KFP decreased from 1,199 kDa to 1,143 kDa after simulated digestion and further decreased to 936 kDa after fermentation	KFP retains in vitro hypolipidemic and hypoglycemic activity after mock digestion	KFP significantly promoted the proliferation of *Anabaena, Lactobacillus* and *Bifidobacterium*, inhibited the growth of *Bilophila*.	The concentration of total SCFAs increased from 9.56 mM to 40.53 mM, with acetic, propionic, and butyric acids as the main components.	(92)
*Clitocybe squamulose*	CSFP	Molecular weight of CSFP decreases from 19,480 kDa to 10,945 kDa during simulated salivary-gastrointestinal digestion	The antioxidant activity of CSFP, and the inhibitory activity of α-glucosidase and α-amylase decreased after digestion in vitro.	CSFP significantly increased the relative abundance of *Bacteroides* and *Prevotella_9,* decreased the abundance of *Escherichia-Shigella.*	CSFP significantly promoted acetic, propionic, and butyric acids, and the content of total SCFAs increased from 2.27 to 35.29 mM, which was significantly higher than the positive control group (INL group, from 2.23 mM to 24.28 mM)	(93)
*coix seed*	CSPW	The molecular weight of CSPW decreased from 30.56 ± 0.31 kDa to stabilize at 23.09 ± 0.14 kDa after simulated digestion.	Functional prediction analysis showed that amino acid metabolism, nucleotide metabolism and carbohydrate metabolism were significantly enhanced by CSPW fermentation.	CSPW was efficiently utilized by the gut microbiota, significantly increasing the abundance of *Lactobacillus-like, Bifidobacterium, Collinsella*, and *Prevotella*, while decreasing the abundance of *Aspergillus*.	CSPW fermentation promoted the production of SCFAs, and the concentration of total SCFAs reached 10.58 ± 0.31 mg/mL, which was significantly higher than that of the blank group (5.49 ± 0.17 mg/mL) and inulin (7.25 ± 0.33 mg/mL)	(94)
*straw mushroom (Volvariella volvacea)*	VVP	The molecular weight of VVP decreased by only 8.9% during simulated digestion and significantly decreased by 40.4% after fecal fermentation	VVP has a slight increase in reducing sugar content during simulated digestion, and some glycosidic bonds may be disrupted	VVP significantly increased the relative abundance of *Bacteroides* and *Phascolarctobacterium*, while decreasing the abundance of *Escherichia-Shigella.*	VVP fermentation significantly promoted acetic, propionic and butyric acids.	(95)
*Imperata cylindrica*	ICP	The molecular weight of ICP decreased slightly from 45.0 kDa to 37.81 kDa in simulated gastric digestion and to 37.53 kDa in simulated enteric digestion	ICP showed significant antioxidant activity during in vitro fermentation process	ICP significantly modulated the composition of the gut microbiota, the abundance of *lactobacillus, Bifidobacterium* and *Roseburia,* while reducing the abundance of harmful bacteria such as *Escherichia-Shigella* and *Helicobacter.*	ICP was efficiently utilized by the gut microbiota, significantly increasing the concentration of acetic, propionic and butyric acids	(96)
*Ziziphus Jujuba* cv. *Pozao*	JPS	The molecular weight of JPS decreased from 1298.85 kDa to 1174.26 kDa	N	JPS significantly increased the number of *Firmicutes, Megasphaera,* and *unclassified_f_Veillonellaceae* and decreased the number of *Bacteroides*, *Proteobacteria, Sutterella,* and *Bilophila.*	The concentration of acetic acid was significantly higher in the JPS group than in the blank and inulin control groups, and butyric acid was significantly higher than in the blank group	(97)
*Agaricus bisporus*	ABP	The antioxidant activity and inhibitory activity against α-glucosidase and α-amylase of ABP changed slightly after digestion	ABP molecular weight remains essentially constant	ABP significantly increased the number of *Bacteroides* and *Prevotella*, while decreasing the number of *Fusobacterium* and *Escherichia.*	ABP fermentation significantly promoted the production of SCFAs, especially acetic, propionic and butyric acids	(98)
*snow chrysanthemum*	JHP	The molecular weight of JHP gradually decreased from an initial 473 kDa to 336 kDa in simulated digestion, and further decreased to 262 kDa in fecal fermentation.	Reducing sugar content increases significantly during digestion in the gastrointestinal tract (11.7-fold increase from the initial value)	JHP increased the relative abundance of beneficial bacteria such as *Bifidobacterium, Lactobacillus, Macrocystis,* and *Macrococcus,* and decreased potentially harmful bacteria such as *Clostridium* and *Aspergillus.*	JHP significantly increased the concentrations of acetic acid, propionic acid and butyric acid, and the total SCFAs concentration reached 23.98 mM after 48 h of fermentation.	(99)
*Pinelliae Rhizoma Praeparatum Cum Alumine*	PPA	PPA exhibits high stability and molecular weight remains essentially unchanged	PPA exhibits significant antioxidant activity during in vitro fermentation processes	PPA significantly increased the abundance of beneficial bacteria such as *Bacteroides* and *Bifidobacterium* and decreased harmful bacteria such as *Desulfobacteriota* and *Enterobacteriaceae.*	Concentrations of acetic acid and propionic acid increased significantly during PPA fermentation, from an initial 8.15 mM and 2.14 mM to 55.78 mM and 4.13 mM after 48 h, respectively.	(103)
*flaxseed*	FSP	FSP was not degraded in the upper gastrointestinal tract and gradually decreased during fermentation in vitro	Reducing sugar content increased significantly at the beginning of fermentation (12 h) and then decreased gradually.	FSP fermentation significantly increased beneficial bacteria such as *Prevotella, Vibrio falciparum, Clostridium* spp. and *Chironobacteria,* while decreasing potentially harmful bacteria such as *Aspergillus*.	FSP fermentation significantly increased the total amount of SCFAs, including acetic, propionic and butyric acids, to 28.52 mM	(104)
*Polygonatum cyrtonema*	PCP	Polygonatum cyrtonema (PCP-80%) is a homogeneous polysaccharide with a molecular weight of 4.1 kDa	PCP has prebiotic activity in regulating gut microbiota and metabolites	PCP significantly promoted the growth of *Megamonas, Bifidobacterium* and *Phascolarctobacterium,* while reducing the relative abundance of *Shigella*.	PCP increased the concentration of total SCFAs after 48 h fermentation to 142.34 mM, especially acetic and propionic acids	(105)
*Helicteres angustifolia L*	HaLPs	HaLPs were not degraded during simulated salivary, gastric and small intestinal digestion, and their molecular weight remained stable.	N	HaLPs significantly promoted the proliferation of beneficial bacteria such as *Anabaena* and *Bifidobacteria,* while reducing the relative abundance of *E. coli.*	The concentration of total SCFAs increased significantly from an initial 2.25 mM to 22.45 mM after 24 h. Acetic acid and propionic acid were the main constituents	(106)
*loquat leaves*	LLP	The molecular weight of LLP-I was 301.4 kDa, which was further reduced to 215.3 kDa after 48 h of fermentation.	The reducing sugar content of LLP gradually increases and the degree of esterification (DE) decreases during digestion in the gastrointestinal tract.	LLP-I significantly increased the phase abundance of *Megasphaera, Megamonas* and *Bifidobacterium* and decreased the abundance of *Fusobacterium* and *Escherichia-Shigella.*	LLP-I promoted the total SCFAs content to 27.01 ± 7.49 mM, which was significantly higher than that of the blank control group (15.88 ± 0.23 mM) and the positive control group (FOS group, 22.95 ± 1.59 mM)	(107)
*Fuzhuan brick tea*	FBTPS-3	Fermentation significantly reduced the molecular weight of FBTPS-3	FBTPS-3 exhibited strong anti-inflammatory activity in inhibiting the LPS-induced inflammatory response in RAW264.7 macrophages.	FBTPS-3 increased the relative abundance of *Bacteroides, arabacteroides* and *Parasutterella*, while decreasing the relative abundance of *Escherichia* and *Shigella*.	FBTPS-3 fermentation significantly increased the levels of SCFAs, especially acetic acid (28.51 ± 3.12 mM), propionic acid and butyric acid	(110)
*Chinese yam*	CYP	The molecular weight of CYP decreased from 254 kDa to 219 kDa during simulated enteric digestion and from 254 kDa to 187 kDa during simulated fermentation.	CYP exhibited anti-inflammatory activity and was able to effectively inhibit the mRNA expression of iNOS, IL-1β and TNF-α and reduce the levels of NO, IL-1β and TNF-α in LPS-stimulated RAW264.7 macrophages.	CYP significantly increased the number of beneficial bacteria (*Bifidobacterium and Megasphaera*) while decreasing the number of harmful bacteria *Escherichia-Shigella.*	The CYP fermentation process increased the concentration of SCFAs, especially the concentration of acetic acid significantly increased from an initial 0.34 mM to 55.36 mM	(111)
*Bletilla striata* polysaccharides	BP	The molecular weight of BP gradually decreased from 221.17 kDa to 21.03 kDa	BP significantly enhances the scavenging of superoxide anion (O2-)	BP significantly increased the abundance of *Veillonella* and *Streptococcus* and decreased the abundance of *Escherichia* and *Bacteroides*	BP significantly contributes to the production of acetic and butyric acids	(112)
*Bletilla striata* oligosaccharides	BO	No significant changes	The scavenging capacity of BO for DPPH radicals was significantly higher than that of BP and controls	BO mainly increased the abundance of *Propionibacterium* and decreased *Escherichia* and *Bacteroides.*	BO mainly promotes the production of propionic acid	(112)
Acetylated *Cyperus esculentus*	ACEPI	Acetylated polysaccharide ACEPI mimics salivary-gastrointestinal digestion after a molecular weight of 56 kDa	ACEPI showed higher antioxidant activity after simulated digestion, with 61.4% DPPH scavenging at 20 mg/mL concentration	ACEPI significantly increased the relative abundance of *Akkermansia* and *Bifidobacterium* and significantly decreased *Proteobacteria* and *Escherichia-Shigella.*	After ACEPI fermentation, the concentration of total SCFAs was significantly higher than that of unacetylated polysaccharide at 34.97 mM	(114)
*Cyperus esculentus*	CEPI	Unacetylated polysaccharide CEPI mimics salivary-gastrointestinal digestion with a molecular weight of 67 kDa	The DPPH clearance of CEPI at a concentration of 20 mg/mL was 52.5% percent	CEPI significantly increased the relative abundance of *Akkermansia* and *Bifidobacterium* and significantly decreased *Klebsiella, Proteobacteria* and *Fusobacterota.*	The total SCFAs concentration after CEPI fermentation was 30.63 mM	(114)
*Boletus mushrooms*	BAP, BBP, and BGP	Boletus polysaccharides exhibited a wide range of molecular weights (5 kDa to 2000 kDa) and the polysaccharide’s composition of monosaccharides gradually decreases during fermentation, especially glucose.	Its biological activity may be closely related to changes in molecular weight and monosaccharide composition	Polysaccharides significantly increased the abundance of *Bacteroides* and *Faecalibacterium* while decreasing the abundance of the harmful bacteria *Escherichia-Shigella* and *Sutterella.*	Increases in acetic and propionic acids were associated with increased abundance of Bacteroides, whereas increases in butyric acid were associated with increased abundance of Firmicutes.	(113)

*Oudemansiella radicata* polysaccharide (ORP) showed a slight decrease in molecular weight from 39.5 kDa to 34.3 kDa in simulated digestion *in vitro*, along with a significant increase in antioxidant activity, presumably related to the decrease in molecular weight and the formation of polysaccharide-protein complexes. During *in vitro* fermentation, ORP was effectively utilized by the gut microbiota, significantly increasing the relative abundance of Bacteroides and Parabacteroides while decreasing the ratio of Firmicutes/Bacteroidetes, suggesting a potential anti-obesity effect (91). In an in vitro simulation of digestion and fermentation, the molecular weight of *kiwifruit pomace* polysaccharide (KFP) decreased from 1,199 kDa to 936 kDa, with a decrease in the content of reducing sugars and a change in the composition of monosaccharides (92). KFP significantly promoted the proliferation of *Bacteroides*, *Lactobacillus,* and *Bifidobacterium,* and at the same time inhibited the growth of harmful bacteria *Bilophila* growth and regulated the balance of gut microbiota. Guo et al. also demonstrated that the molecular weight of *scaly cupuacin* polysaccharide (CSFP) decreased from 19.48 kDa to 10.945 kDa and the content of reducing sugars increased during simulated salivary-gastrointestinal digestion (93). CSFP significantly increased the relative abundance of Bacteroides and Prevotella_9, decreased the abundance of Escherichia-Shigella and improved gut flora structure. *Coix lacryma* polysaccharide (CSPW) showed that the molecular weight of CSPW decreased from 30.56 ± 0.31 kDa to stabilize at 23.09 ± 0.14 kDa after simulated digestion. CSPW was efficiently utilized by the gut microbiota, which significantly increased the numbers of *Lactobacillus* filamentosus-like bacteria, *Bifidobacterium bifidum*, *Collinsella* spp. and *Prevotella* spp., while decreasing the abundance of the phylum Methanogerminae (94). Furthermore, a study has investigated the dynamic changes of physicochemical properties of the grass mushroom polysaccharide (VVP) and its regulation of gut microbiota and metabolites by simulating the salivary-gastrointestinal digestion and *in vitro* fecal fermentation models (95). The results showed that VVP basically remained stable during gastrointestinal digestion, and the molecular weight only decreased by 8.9%, entering the colon as an indigestible polysaccharide. During in vitro fermentation, reducing sugars typically increase initially during hydrolysis before decreasing as they are absorbed by microorganisms. VVP is progressively degraded by gut microbiota, with a significant 40.4% reduction in molecular weight and a marked decrease in reducing sugar content, indicating its effective utilization by gut microbiota.

Polysaccharides with different chemical structures have selective enrichment effects on microbial communities. This structure–function match enables polysaccharides to target and regulate the composition of the flora, inhibiting conditionally pathogenic bacteria while enriching beneficial bacteria such as butyric acid-producing bacteria. Yu et al. showed that *Imperata cylindrica* polysaccharide (ICP) significantly modulated the composition of the gut microbiota, increasing the abundance of beneficial bacteria such as *Lactobacillus, Bifidobacterium* and *Roseburia,* while decreasing the abundance of harmful bacteria such as *Escherichia-Shigella* and *Helicobacter* (96). In addition, molecular docking experiments showed that ICP was able to interact with the key proteins TLR2, MUC1 and MMP9 through high-affinity binding sites. A study systematically investigated the role of *Ziziphus jujuba* cv. *pozao* polysaccharide (JPS) in the regulation of gut microbiota during digestion and fermentation *in vitro*. JPS significantly increased the number of Firmicutes, Megasphaera, and unclassified_f_Veillonellaceae and decreased the number of numbers of *Bacteroides*, *Proteobacteria*, *Sutterella* and *Bilophila* (97). Fu et al. investigated simulated digestion and fecal fermentation modeling and found that *Agaricus bisporus* polysaccharide (ABP) significantly increased the number of *Bacteroides* and *Prevotella*, while decreasing the number of *Fusobacterium* and *Escherichia* (98). During fecal fermentation, snow chrysanthemum polysaccharide (JHP) was further degraded into smaller fragments by gut microbiota, while significantly increasing the relative abundance of beneficial bacteria such as *Bifidobacterium*, *Lactobacillus*, *Macrocystis*, and *Macrococcus*, and decreasing the abundance of potentially harmful bacteria, such as *Clostridium* and *Aspergillus* (99).

#### 4.2.2 Fermented HPs promote the production of SCFAs

Notably, HPs produce a variety of metabolites during *in vitro* fermentation, with the production of SCFAs at the core. Acetic acid, propionic acid and butyric acid play an important role in maintaining intestinal health, as they regulate intestinal pH and inhibit the growth of harmful bacteria, while providing energy to intestinal epithelial cells and promoting the repair and regeneration of intestinal mucosa (100, 101). The analysis of these metabolites can provide insights into the fermentation pathways and mechanisms of polysaccharides, infer the metabolic pathways of polysaccharides under the action of microorganisms, and provide a basis for further research on the prebiotic activity of polysaccharides and their effects on host health (102).

*Pinelliae Rhizoma Praeparatum Cum Alumine* polysaccharide (PPA) showed high stability during simulated digestion. During *in vitro* fermentation, PPA significantly increased the abundance of *Bacteroides and Bifidobacterium,* and the concentrations of acetic acid and propionic acid increased significantly during PPA fermentation, from an initial 8.15 mM and 2.14 mM, respectively, to 55.78 mM and 4.13 mM after 48 h, and these SCFAs play an important role in maintaining intestinal health (103). *Flaxseed* polysaccharide (FSP) was degraded progressively by the gut microbiota, with a gradual decrease in molecular weight and a first increase and then a decrease in reducing sugar content, which was efficiently utilized by the gut microbiota (104). FSP increased the relative abundance of *Prevotella, Vibrio falciparum, Clostridium.* and *Macrocystis*. and decreased the abundance of potentially harmful bacteria such as *Ascomycetes*. at the same time as it simultaneously and significantly increased the total amount of SCFAs (28.52 mM), including acetic, propionic and butyric acids. Another study showed that the polysaccharide derived from *Polygonatum cyrtonema* (PCP-80%) had a molecular weight of 4.1 kDa and consisted mainly of fructose in the *β*-configuration and glucose in the *α*-configuration. During *in vitro* fermentation, pcp-80% increased the production of SCFAs to 142.34 mM, suggesting its prebiotic activity (105). *Helicteres angustifolia L* polysaccharides (HaLPs) significantly promoted the proliferation of beneficial bacteria such as *Anabaena* and *Bifidobacteria*, while reducing the relative abundance of potentially harmful bacteria, and facilitated the increase of total SCFAs from an initial level of 2.25 mM to 22.45 mM after 24 h, suggesting their potential to improve intestinal metabolism and promote host health (106). In addition, Wu et al. investigated the dynamics of physicochemical properties of *loquat* leaf polysaccharides (LLP) and their modulation of gut microbiota and metabolites by simulating salivary-gastrointestinal digestion and *in vitro* cecal fermentation models. LLP-I increased *Megasphaera, Megamonas,* and *Bifidobacterium,* and decreased *Fusobacterium* and *Escherichia-Shigella*. LLP-I fermentation significantly contributed to the total SCFAs content of 27.01 ± 7.49 mM, which is important for gut health (107).

#### 4.2.3 Fermented HPs hold potential for enhancing biological activity

Natural polysaccharides are inherently rich in biological activity, but their activity is further enhanced by structural changes and metabolite generation during *in vitro* simulated digestion and fermentation (108, 109). Specifically, during in vitro digestion and fermentation of polysaccharides, their molecular weight decreases, glycosidic bonds are broken, and the monosaccharide composition may be altered (91). Such structural changes not only increase the solubility and bioavailability of polysaccharides, but also expose more active sites, making it easier for them to interact with biomolecules, thus enhancing bioactivities.

Chen et al. found that Fuzhuan brick tea polysaccharides (FBTPS-3) increased the relative abundance of *Bacteroides*, *Arabacteroides*, and *Parasutterella*, while decreasing the relative abundance of deleterious bacteria (*Escherichia* and *Shigella*), and increased the levels of acetic, propionic, and butyric acids (110). In addition, the fermented FBTPS-3 exhibited strong anti-inflammatory activity in inhibiting the LPS-induced inflammatory response in RAW264.7 macrophages. Meanwhile, the molecular weight of Chinese yam polysaccharides (CYP) remained essentially unchanged in gastric juice but was partially degraded in the small intestine and was progressively decomposed during fermentation. CYP significantly increased the number of *Bifidobacterium* and *Megasphaera*, while decreasing the number of harmful bacteria *Escherichia-Shigella* population (111). Morever, CYP had significant anti-inflammatory and intestinal barrier-protecting effects, especially in the LPS-stimulated co-culture model of Caco-2/RAW264.7 cells that effectively suppressed the inflammatory response. In a comparative study, *Bletilla striata* polysaccharides (BP) and oligosaccharides (BO) exhibited different properties during *in vitro* mock digestion and fermentation (112). the molecular weight of BP gradually decreased from 221.17 kDa to 21.03 kDa, while BO remained stable during mock digestion. During in vitro fermentation, both BP and BO were efficiently utilized by the gut microbiota, with BP significantly increasing the abundance of *Veillonella* and *Streptococcus*, while BO increased the abundance of *Propionibacterium*. Meanwhile, BP significantly promoted the production of acetic acid and butyric acid, while BO mainly promoted the production of propionic acid. In addition, the BP fermentation broth had a higher scavenging capacity for superoxide anion (O2-), while the BO fermentation broth had a higher scavenging capacity for DPPH free radicals. In a study, polysaccharides BAP, BBP, and BGP were extracted from bovine hepatitis bacteria (*B. auripes*, *B. bicolor*, and *B. griseus*) by subcritical water extraction, and *in vitro* fermentation modeling was utilized to explore their regulatory effects on gut microbiota and metabolites (113). It was found that *B. bovis* polysaccharides had a broad molecular weight distribution and a complex monosaccharide composition, with molecular weight gradually decreasing during fermentation and monosaccharides being preferentially utilized by the gut microbiota. BAP improved the composition and structure of gut microbiota, suggesting that it has a significant prebiotic effect and can improve intestinal health by regulating gut microbiota and metabolites. Interestingly, In another comparative study of acetylated polysaccharides, Yuan et al. systematically explored the effects of acetylation of *Cyperus esculentus* polysaccharides on their physicochemical properties, digestive behavior, and intestinal microbial fermentation characteristics (114). Acetylation significantly reduced the molecular weight of the polysaccharide and enhanced its antioxidant activity. In *in vitro* fermentation, the acetylated polysaccharide (ACEPI) exhibited faster fermentation kinetics, produced more SCFAs, and significantly reduced pH. Both polysaccharides modulated the gut microbiota, promoting the growth of the beneficial bacteria *Akkermansia* while inhibiting the harmful bacteria (*Proteobacteria* and *Escherichia-Shigella*). Furthermore, ACEPI significantly increased the abundance of Parabacteroides after fermentation, while CEPI promoted the growth of *Bifidobacterium*. These changes may improve intestinal health and metabolic functions by regulating intestinal metabolites and related protein factors ZO-1 and IL-10, thus demonstrating the potential application of acetylated polysaccharides in functional food development.

Together, these studies show that the fermentation process not only changes the molecular structure of polysaccharides, but also significantly enhances the bioactivity and prebiotic function of polysaccharides by modulating the composition and metabolic activity of the gut microbiota. Collectively, polysaccharides influence microbial interactions and alter the structure and function of microbial communities, which in turn positively affect host health. It is worth noting that experimental results from *in vitro* fermentation modeling need to be interpreted with caution, as they cannot fully mimic the complex physiological environment of the human body (gut motility, host–microbe interactions). Nevertheless, as an efficient and controllable research tool, the in vitro fermentation model provides important theoretical support for exploring the prebiotic activity of HPs and their underlying mechanisms, and lays the foundation for further animal experiments and human clinical trials.

## 5 HPs and their prebiotic activity after fermentation

Prebiotics are indigestible food components that selectively stimulate the growth and activity of beneficial flora in the host’s intestinal tract, thereby beneficially affecting host health (115, 116). HPs and their fermented products exhibit significant bioactivities by regulating the composition and metabolism of the gut microbiota, providing new ideas and strategies for the prevention and treatment of a wide range of chronic diseases (Table 3) (27, 117). A large number of studies have formed a consensus that polysaccharides, in addition to increasing the number and types of beneficial bacteria and improving the microecological balance of the intestinal tract, are also able to regulate the metabolic function of the gut microbiota, affecting their digestion and absorption of nutrients as well as the metabolism of harmful substances (118, 119).

**Table 3:** The prebiotic potential of HPs after fermentation.

Resource	Polysaccharides	Models	Gut microbiota modulation	Healthy benefits	Reference
*Grape seed*	GP	Kunming mice	GP increased the number of *Lachnospiracea_NK4A136_group*, decreased the relative abundance of *Lachnospiracea, Fusobacterium* and *Allisonella.*	GP increased total antioxidant and promoted the production of acetic and propionic acids.	(120)
*Inonotus obliquus*	IN	DSS-induced C57BL/6 mice	Increase *Coriobacteriaceae Bacteria,* and *Firmicutes,* decrease *Odoribacter, Clostridium, Parabacteroides and Turicibacter.*	Increased the microbiota diversity, modulated metabolites involved in aromatic amino acid metabolism, the citrate cycle, purine metabolism, pyrimidine metabolism.	(109)
*Hericium erinaceus*	HEP10	DSS-induced C57BL/6 mice	Increase *Akkermansiamuciniphila, and* decrease *Proteobacteri.*	Suppressed the production of TNF-α, IL-1β, IL-6, inducible iNOS, and COX-2, suppressed the activation of NLRP3 inflammasome, NF-κB, AKT, and MAPK pathways.	(129)
*Ganoderma lucidum*	GLP	4 T1-breast cancer Balb/c mice	Increase *Bacteroides* and *Ruminococcus.*	Recovered the impaired TILs via down-regulating the co-inhibitory signaling, regulated gut microbiota and tumor metabolism.	(131)
*Ganoderma lucidum*	SGP	AOM/DSS-induced cancer Balb/c mice	Increase *Bacteroides, Parabacteroides, Peptostreptococcaceae, Enterobacteriaceae* and *Sutterella,* decrease *Desulfovibrionaceae, Akkermansia,* and *Ruminococcus.*	Decreased concentration of serous TNF-α, improved colon status, upregulated 6 genes in host epithelial cells, including Il10, Cytl1, Igkv7-33, Ighv1-14, Igfbp6 and Foxd3 and altered microbial metabolic pathways.	(130)
*Dictyophora indusiata*	DIP	Clindamycin induced- AAD BALB/c mice	DIP reversed the dysbiosis and increased beneficial flora, including *Lactobacillaceae* (lactic acid-producing bacteria), and *Ruminococaceae* (butyrate-producing bacteria).	It resulted in the reduction of endotoxemia (through LPSs) and pro-inflammatory cytokine (TNF-α, IL-6, and IL-1β) levels, with the increased expression of tight-junction associated proteins (claudin-1, occludin, and zonula occludens-1).	(123)
*Panax ginseng*	WGP	LH induced- AAD Balb/c mice	WGP increased the phylum Firmicutes and decreased the phyla Bacteroidetes, Proteobacteria and Actinobacteria, and increased the relative abundance of *Lactobacillus, Lactococcus,* and *Streptococcus,* but decreased the relative abundance of Bacteroides at the genus level.	It reversed carbohydrate, amino acid and energy metabolism to normal levels, thereby promoting the recovery of the mucosal structure.	(124)
*Bamboo Shoot Byproducts*	BW	LH induced- AAD Kunming mice	BSP affected the composition and diversity of the gut microbiota in the AAD mice, increased the beneficial bacteria such as *Bacteroides, Lactobacillus* and *Lachnospiraceae_NK4A136_group.*	The mice fed with BSP exhibited significant higher bodyweight gain, lower pH value and higher concentrations of SCFAs in the feces, and it reduced the inflammatory cells in the small intestine and colon.	(125)
*Purple Sweet Potato*	PSPP	LH induced- AAD Balb/c mice	PSPP increased the abundance of Bacteroides, and reduced the abundance of *Enterobacteriaceae* and *Klebsiella*	It decreased the levels of IL-1β, IL-6 and TNF-α, reduced the cecal index, improved the ileum tissue morphology, and alleviated the inflammatory response, and increased the content of SCFAs.	(126)
*Poria cocos*	PCY	LH induced- AAD C57BL/6 mice	PCY changed the structure of gut microbiota by increasing the relative abundance of *norank_f__Muribaculaceae* and *unclassified_f__Lachnospiraceae*, and decreasing that of *Escherichia-Shigella, Staphylococcus* and *Acinetobacter.*	It restored the intestinal barrier function, improved the content of SCFAs (acetic and butyric acid), decreased the level of inflammatory cytokines (TNF-α, IL-6 and IL-1β).	(127)
*Poria cocos*	WIP	HFD-induced ob/ob mice.	Increase *Lachnospiracea* and *Clostridium*	Elevated the level of butyrate in gut, improved the gut mucosal integrity and activated the intestinal PPAR-*γ* pathway. Improved glucose and lipid- metabolism, and alleviated hepatic steatosis.	(132)
*Agrocybecylindracea*	ACP	HFD-induced C57BL/6 J mice	Increase *Bacteroides, Parabacteroides, Butyricimonas,* and *Dubosiella,* decrease *Desulfovibrio* and *Oscillibacter.*	Reduced body weight, adipose accumulation, impaired insulin resistance, lipid levels, liver injuries, and pro-inflammatory factors, positively regulated gut microbiota and gut metabolites	(28)
*Lyophyllumdecastes*	LDP	HFD-induced obese mice	Increase *Bacteroides* and *Lactobacillus johnsonii*, decrease *B. vulgatus, B. thetaiotaomicron, B. sartorii, Parabacteroides goldsteinii,* and *P. distasonis*	Ameliorated HFD-induced obesity, hyperlipidemia, and inflammation, up-regulating the secondary bile acids-and activating TGR5 signaling pathway	(133)
*Morchella esculenta*	MEP	HFD-induced T2DM BALB/c mice	Increase *Lactobacillus, decrease Actinobacteria, Corynebacterium, and Facklamia.*	Reduced endotoxemia and insulin resistance-related proinflammatory cytokines, improved intestinal permeability and altered the functional metabolic Pathways to ameliorate T2DM.	(134)

After simulated salivary-gastrointestinal digestion and *in vitro* fecal fermentation, grape seed polysaccharides (GP) extracted by aqueous alcoholic precipitation increased total antioxidant, increased the relative abundance of *Lachnospiracea*_NK4A136_group (*p* = 0.008239), decreased the relative abundance of *Lachnospiracea* relative abundance of *Fusobacterium* and *Allisonella*, and promoted acetic and propionic acids (120). In addition, *Aloe vera* polysaccharides (APs) and soybean polysaccharides (SP) also increased the concentration of SCFAs during in vitro fermentation in a simulator of the human gut microbial ecosystem (SHIME), increasing the concentration of *Bifidobacterium and Lactobacillus*, while decreasing *Escherichia-Shigella* and *Escherichia coli* (121, 122).

The integrity of the intestinal barrier is essential for maintaining intestinal health. HPs are able to enhance the intestinal barrier function and protect the mucosa from damage by regulating the composition and metabolism of gut microbiota. For example, *Dictyophora indusiata* polysaccharides (DIP) were able to reverse clindamycin- and metronidazole-induced intestinal dysbiosis, increase the number of beneficial flora such as *Lactobacillus* (lactic acid-producing bacteria) and *Rumatococcaceae* (butyric acid-producing bacteria), and increase the expression of tight junction-associated proteins, decreasing endotoxemia and proinflammatory cytokine Levels (123). In addition, *Panax ginseng* (WGP) polysaccharide was able to alleviate lincomycin-induced antibiotic-associated diarrhea (AAD) in mice by increasing the number of *Lactobacillus, Lactococcus*, and *Streptococcus*, regulating carbohydrate, amino acid, and energy metabolism, and promoting the restoration of intestinal mucosa (124). *Bamboo shoot* polysaccharide (BW) affected the composition and diversity of the gut microbiota of AAD mice by increasing beneficial bacteria such as *Bacteroidetes mimicus, Lactobacillus* and *Lachnospiraceae*_NK4A136_group (125). Purple Sweet Potato polysaccharide (PSPP) improves gut health in AAD mice by increasing *anaplasmosis*, decreasing *Enterobacteriaceae* and *Klebsiella pneumoniae*, decreasing inflammation levels and cecum index, and improving ileal tissue morphology (126). It is worth noting that *Poria cocos* (PCY)-derived water-insoluble polysaccharides were also effective in alleviating the symptoms of lincomycin hydrochloride-induced AAD after fermentation. PCY increased the number of *Muribaculaceae* and *Lachnospiraceae,* and decreased the number of *E. coli-Shigella, Staphylococcus,* and *Fusobacterium* (127).

Extensive studies have demonstrated that prebiotics can degrade complex plant polysaccharides into oligosaccharides or monosaccharides during fermentation by secreting a series of carbohydrate-active enzymes (CAZymes), such as *β*-galactosidase, *α*-glucosidase, and arabinase. This process subsequently produces acetic acid, propionic acid, and butyric acid (19). Regarding immunomodulation, SCFAs produced by *Lactobacillus* and *Bifidobacterium* through polysaccharide fermentation promote regulatory T cell (Treg) differentiation and suppress Th17 cell-mediated inflammatory responses, thereby maintaining intestinal immune homeostasis. Furthermore, these strains degrade polysaccharides to generate immunologically active oligosaccharide fragments that directly activate macrophages and dendritic cells. This enhances expression of anti-inflammatory factors like IL-10 and IFN-*γ*, thereby bolstering host immune defense capabilities (128).

Inflammatory response is an important pathological feature of many chronic diseases, and gut microbiota plays a key role in regulating the inflammatory response. HPs and their fermentation products can significantly decrease inflammatory markers and mitigated symptoms by modulating the composition and metabolism of gut microbiota. For example, *Inonotus obliquus* polysaccharide (IN) significantly ameliorated DSS-induced colitis in a DSS-induced C57BL/6 J mouse model by increasing gut microbial diversity and modulating systemic metabolism (109). In addition, *Hericium erinaceus* low molecular mass polysaccharide (HEP10) significantly ameliorated DSS-induced colitis by inhibiting the induction of pro-inflammatory cytokines iNOS and cyclooxygenase-2 (COX-2), as well as inhibiting the activation of gut flora-associated NLRP3 inflammasome vesicles (129).

HPs play an important role in immunomodulation and can regulate the body’s immune response through multiple pathways. On the one hand, it can activate immune cells, such as macrophages, T-cells and B-cells, and enhance their phagocytosis, cytokine secretion and immune response. On the other hand, polysaccharides can induce macrophages to release cytokines such as tumor necrosis factor-*α* and interleukin-1, which can enhance the body’s inflammatory response and immune defense ability. For example, *Ganoderma lucidum* spore polysaccharide (SGP) was able to ameliorate AOM and DSS-induced male AOM and DSS-induced male by decreasing the concentration of TNF-α, up-regulating the expression of genes such as Il10, Cytl1, Igkv7-33, Ighv1-14, Igfbp6, Foxd3, and altering the metabolic pathways of intestinal microorganisms in the host epithelial cells. Colon cancer in Balb/c mice (130). Furthermore, *Ganoderma lucidum* polysaccharide (GLP) inhibited colonic inflammation and tumorigenesis by modulating gut microbiota and immune cell function in AOM/DSS-induced C57BL/6 mice, as evidenced by increased production of SCFAs, attenuation of endotoxemia, down-regulation of IL-1β, iNOS, and COX-2 expression, and inhibition of lipopolysaccharide (LPS) induced inflammatory markers and MAPK activation (131). In clinical applications, the use of nutraceuticals or drugs containing HPs in immunocompromised patients can effectively improve the body’s immunity and reduce the risk of infectious diseases.

Accumulating evidence indicates shows that HPs have a positive impact on metabolic health. In terms of blood glucose regulation, some polysaccharides can slow down the digestion and absorption of carbohydrates by inhibiting the activities of *α*-amylase and α-glucosidase, thus lowering the rise of blood glucose after meals. In lipid regulation, HPs can lower the levels of total cholesterol, triglyceride and LDL cholesterol in blood, and at the same time raise the level of HDL cholesterol, which can help prevent and improve cardiovascular diseases such as atherosclerosis. For example, *Agrocybe cylindracea* polysaccharide (ACP) reduced the number of *Vibrio desulfuricans*, increased the abundance of *Vibrio parahaemolyticus*, and decreased the levels of TNF-α and IL-6 in mice induced by a high-fat diet (28). The water insoluble polysaccharide of *Poria cocos* (WIP) significantly reduced obesity symptoms by increasing butyric acid-producing bacteria and butyric acid levels, improving intestinal mucosal integrity, activating the intestinal PPAR-*γ* signaling pathway, and improving glucose and lipid metabolism (132). *Lyophyllum decastes* polysaccharide (LDP) significantly ameliorated high-fat diet-induced obesity, hyperlipidemia, and inflammation by modulating gut microbiota, increasing the upregulation of secondary bile acids, and activating the TGR5 signaling pathway (133). In addition, *Morchella esculenta* polysaccharide (MEP) significantly ameliorated high-fat diet-induced diabetes by augmenting *Lactobacillus*, inhibiting enterococci, and modulating inflammatory cascade responses, including Toll-like receptor 4 (TLR4), COX-2, and NF-κB (134). Without exception, these studies have shown that HPs can have a beneficial impact on overall metabolic health by regulating gut flora and their metabolites and improving energy metabolism and fat storage.

## 6 Conclusions and outlooks

Emerging evidence highlights a surge of interest in fermented HPs. This review synthesizes current evidence that microbial fermentation, mediated by either specific bacterial strains or complex fecal consortia, remodels the native plant polysaccharides, markedly enhancing and diversifying their biological activities. In individuals with intestinal dysbiosis, supplementation with targeted HPs preferentially promotes the proliferation of beneficial gut commensals, restoring microbial equilibrium and improving overall gastrointestinal health. However, limitations lie in the fact that we have not yet fully integrated the “polysaccharide-enzyme-metabolite-receptor,” nor have we conducted direct validation using gene knockout and germ-free animals. Moreover, the *in vivo* mechanisms of polysaccharides are complex and influenced by various factors, and it is challenging to deeply investigate their targets and signaling pathways. As researchers continue to explore, new analytical techniques, synthetic methods, and research approaches are opening up greater possibilities for in-depth studies of polysaccharides. Based on the *in vitro* fermentation characteristics of HPs and their impact on the gut microbiota, there is potential to develop innovative drugs for the treatment of immune-related diseases, metabolic disorders, and cancer. This review examines the impact of fermented HPs on microbial communities and their biological effects, providing a theoretical foundation for the effective utilization of herbal medicines and insights into the development of novel functional products. Future research should combine dynamic fermentation models with stratified population trials to further advance the translation of herbal polysaccharides from laboratory studies to clinical applications.

## Glossary

AAD: Antibiotic-associated diarrhea
ACEPI: Acetylated *Cyperus esculentus* polysaccharide
ACP: *Agrocybe cylindracea* polysaccharide
AF4: Asymmetric flow field-flow fractionation
ABP: *Agaricus bisporus* polysaccharide
BRP2-2: *Brassica rapa* L. polysaccharide fraction 2–2
CAZymes: Carbohydrate-active enzymes
CEPI: Native *Cyperus esculentus* polysaccharide
C0X-2: Cyclooxygenase-2
CYP: Chinese yam polysaccharide
DIP: *Dictyophora indusiate* polysaccharide
EPS: Extracellular polysaccharides
FBTPS-3: Fuzhuan brick tea polysaccharide fraction 3
FVP: *Flammulina velutipes* polysaccharide
GLP: *Ganoderma lucidum* polysaccharide
GP: Grape seed polysaccharide
HEP10: *Hericium erinaceus* low-molecular-mass polysaccharide
HPs: Herbal polysaccharides
ICP: *Imperata cylindrica* polysaccharide
IN: *Inonotus obliquus* polysaccharide
JPS: *Ziziphus jujuba* cv. Pozao polysaccharide
KFP: Kiwifruit pomace polysaccharide
LLP: Loquat leaf polysaccharide
LPF: *Lactobacillus fermentum* polysaccharide
LPS: Lipopolysaccharide
MALDI-TOF MS: Matrix-assisted laser desorption/ionization time-of-flight mass spectrometry
MALLS: Multi-angle laser light scattering
MEP: *Morchella esculenta* polysaccharide
NCVP: *Nostoc commune* Vauch. polysaccharide
ORP: *Oudemansiella radicata* polysaccharide
PCP: *Polygonatum cyrtonema* polysaccharide
PKPS: *Polygonatum kingianum* polysaccharide
PPA: *Pinelliae Rhizoma Praeparatum Cum Alumine* polysaccharide
PSPP: Purple sweet potato polysaccharide
RI: Refractive index
SC: Size-exclusion chromatography
SCFA: Short-chain fatty acid
SGP: *Ganoderma lucidum* spore polysaccharide
SHIME: Simulator of the human intestinal microbial ecosystem
THDP2: *Tetrastigma hemsleyanum* polysaccharide fraction 2
TLR4: Toll-like receptor 4
VVP: *Volvariella volvacea* polysaccharide
WGP: *Panax ginseng* polysaccharide
WIP: Water-insoluble *Poria cocos* polysaccharide
